# Performance and Kinetics Study of Self-Repairing Hydroxyl-Terminated Polybutadiene Binders Based on the Diels–Alder Reaction

**DOI:** 10.3390/polym9060200

**Published:** 2017-05-30

**Authors:** Chuyao Liang, Jie Li, Min Xia, Guoping Li, Yunjun Luo

**Affiliations:** School of Materials Science and Engineering, Beijing Institute of Technology, Beijing 100081, China; growean_leong@163.com (C.L.); leejie@bit.edu.cn (J.L.); girlping3114@bit.edu.cn (G.L.)

**Keywords:** Diels–Alder reaction, hydroxyl-terminated polybutadiene, binder, self-repairing, solid propellant

## Abstract

Based on the Diels–Alder reaction and hydroxyl-terminated polybutadiene (HTPB) binders of solid propellants, two novel compounds—furfuryl-terminated polybutadiene (FTPB) and trifurfuryl propane (TFP)—were designed and synthesized, and their structures were characterized. A new kind of reversible Diels–Alder reaction system was formed by FTPB as main resin, *N*,*N*′-1,3-phenylenedimaleimide (PDMI) as curing agent and TFP as chain extender. The results showed that this system had good mechanical properties with a tensile strength of 1.76 MPa and a tensile strain of 284% after curing, and the repair efficiency of the crack was 88%. Therefore, it could be used as a novel binder of energetic materials such as solid propellant and PBX explosives to provide them with self-repairing characteristics and improve the reliability for application.

## 1. Introduction

The ability of the damaged organisms responding appropriately to restore their own performance, which is termed as self-repairing, has been used in material applications. Based on whether the repair agent is used, the self-repairing process of polymer materials can be divided into extrinsic and intrinsic methods [1]. The extrinsic repair method uses microcapsules [2,3] and hollow fibers [4,5] to contain restorative agents. The intrinsic repair method is self-repairing using the reversible chemical reactions present in the reactive system, and the reversible chemical reactions include Diels–Alder reaction [6], dynamic covalent chemistry [7,8], disulfide bond reaction [9,10], coordination [11], hydrogen-containing supramolecular structure [12], and so on.

However, when a binder for the energetic material is affected by heat as well as mechanical and chemical factors, the defects of the microcracks may be generated inside the energetic material, which can have a significant effect on the mechanical properties and application reliability of the energetic materials. So, it has a good application prospect to introduce self-repairing properties into the energetic materials.

Hydroxyl-terminated poly-butadiene (HTPB) can be cured by isocyanate-based curing agent with good mechanical properties. It is the most commonly used binder of energetic materials such as composite solid propellant, PBX explosive, gas generating agent, and many more [13]. HTPB binders have polyhydroxy functional groups and thereby provide the energetic material with a certain shape and certain mechanical properties. If the HTPB binder system could be self-repairing, it is expected to significantly improve the application reliability of the damaged energetic materials and prolong the service life.

Due to the addition of repair agents tending to affect the mechanical and energy properties, and the prohibition to use on the repair conditions such as ultraviolet light and electric field when energetic materials containing metal component charge, so the Diels–Alder reaction is more suitable for constructing a reversible cross-linked repair reaction in energetic materials applications. The Diels–Alder reaction [6,14,15,16] is a thermoreversible reaction whose monomer involved in the reaction is simple to synthesize and can be controlled by temperature without the addition of a catalyst. For example, Wudl [17] prepared a “dynamic cross-linking” furan-maleimide structure on the base of positive and inverse Diels–Alder reaction at different temperatures. As a repair unit, a furan-maleimide structure can break and recombine the cross-linked structure through the thermal process to complete the repair of microcracks.

In this paper, the Diels–Alder reaction was introduced into the HTPB binder, the synthesis of furfuryl-terminated polybutadiene (FTPB) and trifurfuryl propane (TFP) was carried out, and the forward reaction kinetics was studied. It is expected to provide a new kind of self-repairing binder for energetic materials such as solid propellants.

## 2. Experimental

### 2.1. Materials

Hydroxyl-terminated polybutadiene (HTPB) was obtained from the Liming Chemical Research Institute (Luoyang, China), whose *M*_n_ is 3000 g/mol and hydroxyl value is 0.75 mmol/g, 2-furoic acid (FA) was obtained from Sinopharm Chemical Reagent Co. Ltd. (Beijing, China), 4-dimethylaminopyridine (DMAP, ≥99%) was purchased from Aladdin Industry Corporation (Beiing, China), dicyclohexylcarbodiimide (DCC, ≥99%) and trimethylolpropane (TMP, ≥98%) was purchased from Tianjin Guangfu Fine Chemical Research Institute (Tianjin, China), and *N*,*N*′-1,3-phenylenedimaleimide (PDMI, ≥97%) was purchased from J&K Scientific Ltd. (Beijing, China).

### 2.2. Characterization

Fourier transform infrared (FTIR) spectra were recorded with Nicolet 8700 infrared spectrometer from Thermo Nicolet Corporation (Waltham, MA, USA). The experimental conditions were as follows: The number of scans is 48 times per minute, the resolution is 4 cm^−1^, and the scanning range is from 400 to 4000 cm^−1^.

Hot stage polarized microscope (HSPM) thermograms were performed with DM2500P polarized microscope from Leica Corporation (Wetzlar, Hesse, German) using a 12 V 100 W Halogen lamp under temperatures from 60 to 100 °C.

Tensile performance was tested by AGS-J electronic universal testing machine of Shimadzu Corporation (Kyoto, Japan), the tensile rate of 100 mm/min at 25 °C, according to GB/T528-1998 method to prepare the sample into a dumbbell-shaped spline.

Scanning electron microscopy (SEM) images were recorded by an S-4800 field emission scanning electron microscope of the Hitachi Corporation (Tokyo, Japan) at a 2.0 kV acceleration voltage and an 8 mm beam spot at a magnification of 3000.

UV-Vis test was performed by U-3010 spectrophotometer of Hitachi Corporation (Tokyo, Japan), and the test wavelength ranged from 200 nm to 800 nm.

### 2.3. Synthesis of FTPB

Hydroxyl-terminated polybutadiene esterified with 2-furoic acid at 25 °C in dichloromethane, wherein DMAP was used as an esterification catalyst [18], DCC was used as a dehydrating agent [19], and the by-product 1,3-dicyclohexylurea (DCU) generated from DCC after water absorption was insoluble in dichloromethane, so that the reaction solution was a milky white turbid liquid. After filtration to remove impurities, the brown liquid could be obtained and the filtrate was then kept at −10 °C for 6 h, and the precipitation of the residual DCC could also be removed by filtration to attain a clearer filtrate. The filtrate, which could be washed by distilled water to remove DMAP, was ultimately separated by the column chromatography whose elute was a 1:20 ratio of ethyl acetate: petroleum ether in “R” value of about 0.5. After removing the solvent by rotary evaporation at 40 °C, the pure furfuryl terminated polybutadiene could be obtained, namely FTPB, and the product was a light yellow transparent liquid with a yield of 57%.

### 2.4. Synthesis of TFP

**Figure 1:**
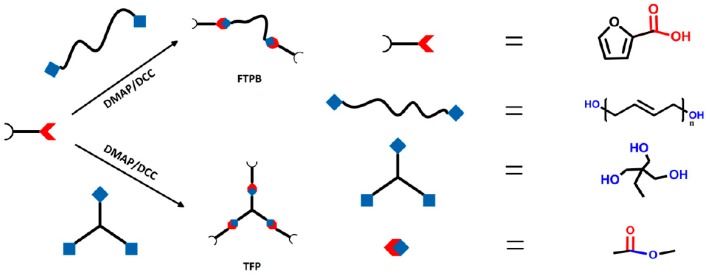
Synthesis routes of furfuryl-terminated polybutadiene (FTPB) and trifurfuryl propane (TFP).

The synthesis routes of FTPB and TFP are both shown in Figure 1. As the curing agent PDMI is bifunctional, FTPB with a functionality of slightly greater than 2 is dominated by the linear network, which affects the binder’s mechanics performance. Therefore, it is necessary to introduce a 3-functional chain extender TFP to modify the cross-linked network structure.

Trimethylolpropane was esterified with 2-furoic acid at 25 °C in dichloromethane, wherein DMAP was used as an esterification catalyst, and DCC was used as a dehydrating agent, to obtain the target product of trifurfuryl propane (TFP) with a yield of 63%.

### 2.5. Preparation of Self-Repairing Binder System

Furfuryl-terminated polybutadiene (FTPB) was added dropwise into a solution of *N*,*N*′-1,3-phenylenedimaleimide (PDMI) dissolved in dichloromethane, and ring-forming Diels–Alder reaction carried out at 60 °C for 2 h to complete the preparation of prepolymer.

TFP, dissolved in a few dichloromethane, was added into the FTPB-PDMI prepolymer. After homogeneous mixing, the dichloromethane was removed by vacuum under room temperature, and the post-extended chain reaction was completed after curing at 60 °C for 48 h to obtain the prepared sample.

## 3. Results and Discussion

### 3.1. Structure Characterization of FTPB and TFP

**Figure 2:**
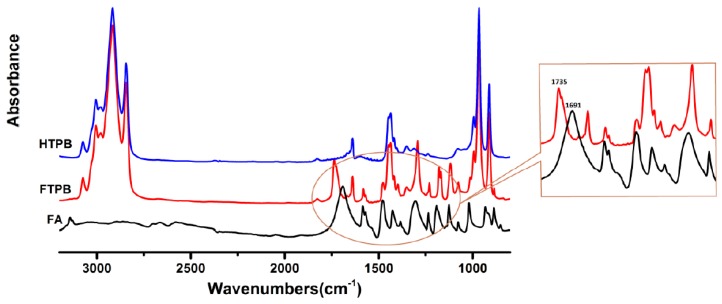
FT-IR spectrum of the comparison among hydroxyl-terminated polybutadiene (HTPB), FTPB, and furoic acid (FA): The peaks of furoic groups could be seen at 1730 cm^−1^ (C=O bonds stretching), 1290, 1180, and 1120 cm^−1^ (furan rings stretching), 1580, 1480, and 1350 cm^−1^ (C=C bonds stretching), 1230 and 1080 cm^−1^ (C–O–C bonds stretching), and 762 cm^−1^ (furoic C–H bonds out-of-plane bending).

Figure 2 is the FTIR spectrum of the comparison among HTPB, FTPB, and FA. From Figure 2, distinguished absorption peaks of ester carbonyl and furan ring could be observed in the FTIR spectrum of FTPB contrasted with HTPB and FA: the carbonyl of furoic acid peak shifted from 1691 to 1735 cm^−1^ after esterification, which lent sufficient support to the accomplishment of esterification reactions.

**Figure 3:**
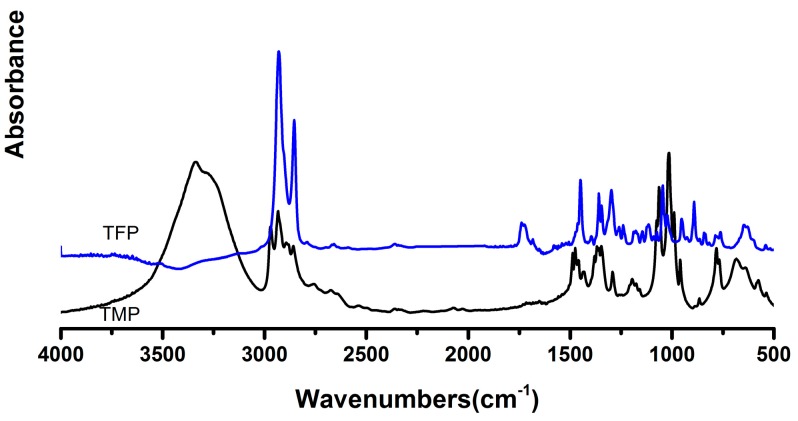
FT-IR Spectrum of the comparison between trimethylolpropane (TMP) and TFP: the peaks of furoic groups were showed at 1734 cm^−1^ (C=O bonds stretching), 1297, 1181, and 1117 cm^−1^ (furan rings stretching), 1449 and 1350 cm^−1^ (C=C bonds stretching), 1238 and 1045 cm^−1^ (C–O–C bonds stretching), and 763 cm^−1^ (furoic C–H bonds out-of-plane bending).

**Figure 4:**
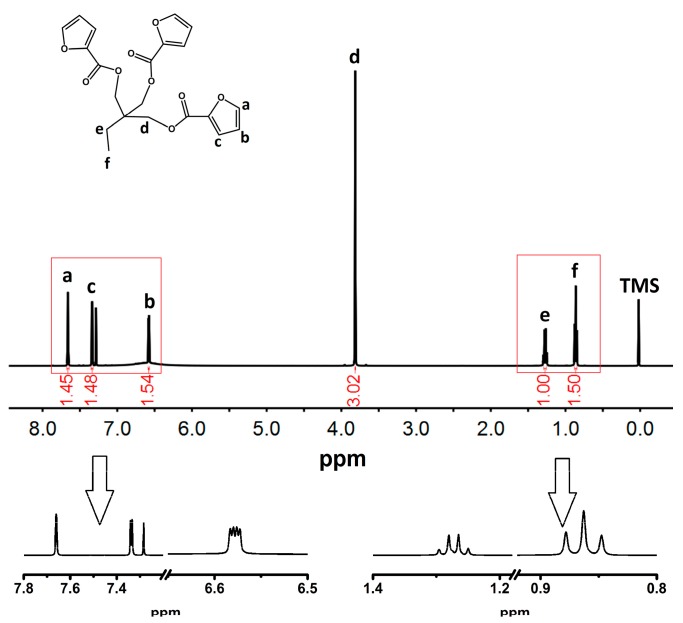
^1^H nuclear magnetic resonance (NMR) spectrum of TFP: δ = 7.66, 3H; δ = 7.32–7.34, 3H; δ = 6.57–6.59, 3H; δ = 3.81, 6H; δ = 1.25–1.30, 2H; δ = 0.85–0.88, 3H. FTIR on FTPB-PDMI film crack self-repairing progress. PDMI: *N*,*N*′-1,3-phenylenedimaleimide.

In the FTIR (KBr, cm^−1^) spectra of TFP and TMP (Figure 3), it was also found that the absorption peaks of the furan group and the carbonyl group were shown to fully prove that the furoic acid groups were successfully connected to the ends of TMP. Moreover, the results of ^1^H NMR of TFP shown in Figure 4 revealed coincidence with the number of hydrogen atoms contained in the monomer, further supporting the chemical structures of FTPB.

**Figure 5:**
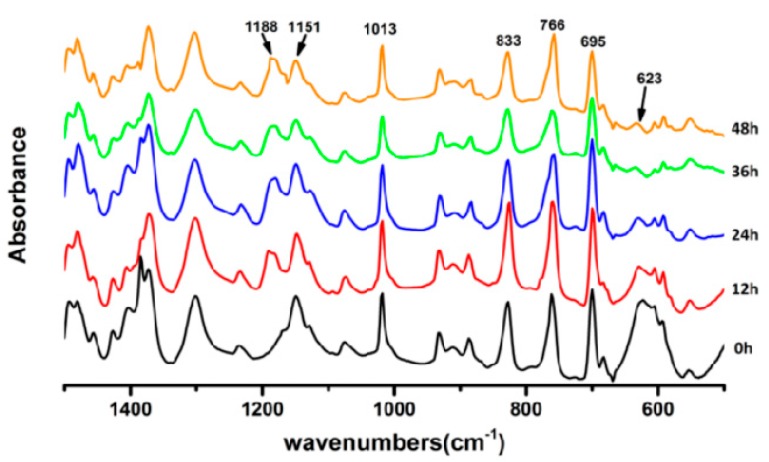
The infrared absorbance of FTPB-PDMI’s functional group with the reaction time (the stretching vibration of furan rings corresponding to the peaks at 1151 and 1013 cm^−1^; the peaks at 1188 cm^−1^ corresponding to the C–C bonds’ stretching vibration of Diels–Alder adducts; the peaks at 833, 766, and 695 cm^−1^ corresponding to out-of-plane bending vibration of C–H bonds on C–C bonds; the peaks at 623 cm^−1^ corresponding to the out-of-plane bending vibration C–H bonds on C=C bonds of maleimide).

In order to investigate whether the microcrack’s self-repairing process of the film is the process of Diels–Alder reaction or the process of the thermoplastic transformation, the qualitative characterization of the self-repairing process was carried out by FTIR as shown in Figure 5. On the base of the stretching vibration of furan rings’ peak at 1013 cm^−1^, it can be easily figured out that there is no characteristic absorption peak at 1188 cm^−1^ on 0 h. With the increase in reaction time, the absorption peak occurs and gradually increases, corresponding to the absorption vibration peak of C–C in the Diels–Alder adduct, which shows that furan group and malaimide group undergoes a Diels–Alder reaction; the peaks at 623 cm^−1^ are caused by the out-of-plane bending vibration of C–H bonds on C=C bonds of maleimide, which almost completely disappeared at 48 h, indicating that the PDMI monomer was gradually consumed with the increase in reaction time.

### 3.2. Mechanial Property of FTPB/PDMI/TFP (FPT) Film

**Figure 6:**
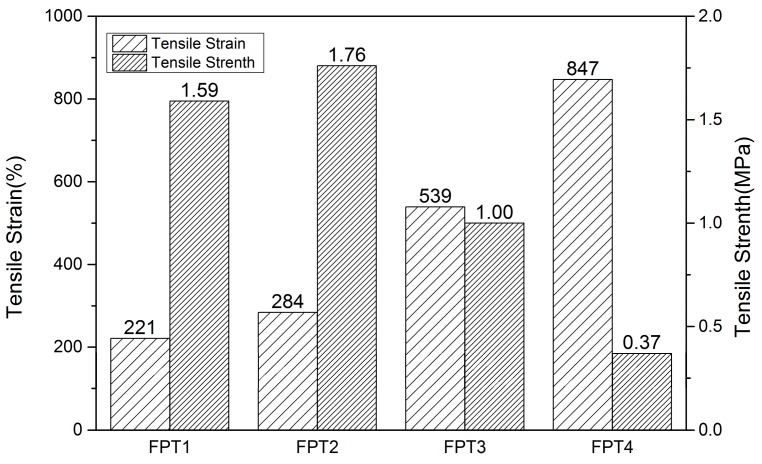
The effect of different chain-extension ratios on tensile strain and strength of films.

Low bond energy of the Diels–Alder bonds in the material will lead to the decline of mechanical properties, which is the same to the FTPB-PDMI system. It is not enough to maintain the mechanical performance only by the FTPB-PDMI system, so this paper will use the FTPB-PDMI system of Diels–Alder reaction as the main system with a certain proportion of TFP as a chain extender to improve mechanical property and self-repairing performance. The tensile performance of the FTPB film only cured by PDMI was tested, and the tensile strain and tensile strength data is shown in Table 1: the mechanical strength of the film was low. The addition amounts of the chain extender TFP were, separately, 20%, 10%, 5%, and 0%, and corresponding samples FPT1, FPT2, FPT3, and FPT4, respectively, were obtained and their tensile properties are shown in Figure 6.

It could be investigated that the tensile strength of the film was improved remarkably after TFP added, and the tensile strength was 1.76 MPa at the addition amount of chain extender of 10%, and it was nearly 5 times higher than the tensile strength of 0.37 MPa without chain extension. Because the addition of TFP could improve the degree of crosslinking, the degree of intermolecular entanglement, and the molecular chain rigidity, so that the mechanical properties were improved. However, the tensile strength decreased at addition amounts of the chain extender ratios greater than 10%, and the tensile strain also showed a tendency to gradually decrease, for that the film is less prone to brittle fracture than the low cross-linked film with the increase in the degree of cross-linking.

A sharp pre-crack was created in the FPT1, FPT2, and FPT3 by gently tapping a fresh razor blade [17], which was heated at 120 °C for 2 h and then cured at 60 °C for 48 h. The tensile properties of the three splines with no cracks samples (FPT1, FPT2, and FPT3) and pre-crack repair samples are shown in Table 1, and the repair efficiency of the film was quantitatively analyzed from the two parameters of tensile strength and tensile strain.

Compared with three different addition ratios of the films, FPT1 and FPT3 had lower repair efficiencies than FPT2 in the tensile strain, but the tensile strength repair efficiency of FPT1 was improved. As the linear structure was conducive to the extension of the molecular chain, the network structure was conducive to increasing the molecular chain rigidity, so as the chain expansion ratio increases, the network structure was easier to form the linear structure after fracture, which was favorable to the extension of the molecular chain, resulting in an increase of the elongation at break; simultaneously, after the linear structure was broken, it was easier to repair with the network structure to improve the molecular chain rigidity and reduce the tensile strength repair efficiency.

### 3.3. HSPM on FTPB-PDMI Film Crack Self-Repairing Progress

In order to study the self-repairing condition of the microcracks, this chapter will characterize the self-repairing performance of the hot stage polarized microscope (HSPM) and the tensile strength before and after film repair.

**Figure 7:**
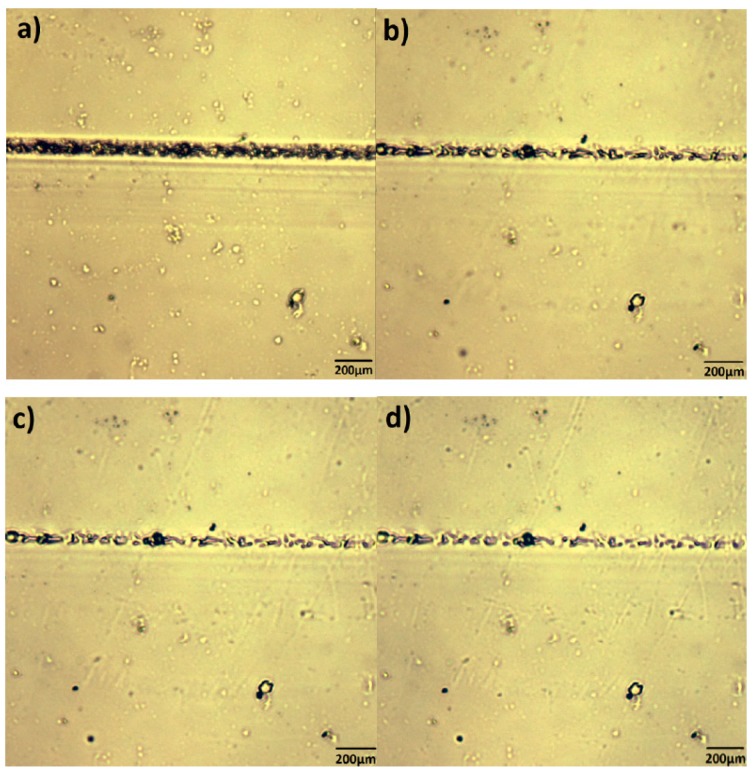
Hot stage polarized microscopic images of (**a**) knife-cutting film; (**b**) film after repairing at 60 °C for 24 h; (**c**) film after repairing at 60 °C for 48 h; (**d**) film after repairing at 60 °C for 72 h.

The self-repairing progress of FPT2 cross-linked material was observed by HSPM, and we could intuitively find out that prefabricated cracks are clearly repaired (Figure 7) after heating at 60 °C for 72 h. Above all, the film with a crack was placed at 120 °C for 2 h (Figure 7a), debonding occurred to provide an ability of chain reformation, and then placed at 60 °C for 48 h (Figure 7b,c), a clearly recovered sample was obtained, and the crack was gradually restored to the original surface. The width was obviously reduced compared with that before repairing, indicating that the film had self-repairing properties. When the film with crack was heated to 72 h at 60 °C, the crack did not show any significant change (Figure 7d).

**Figure 8:**
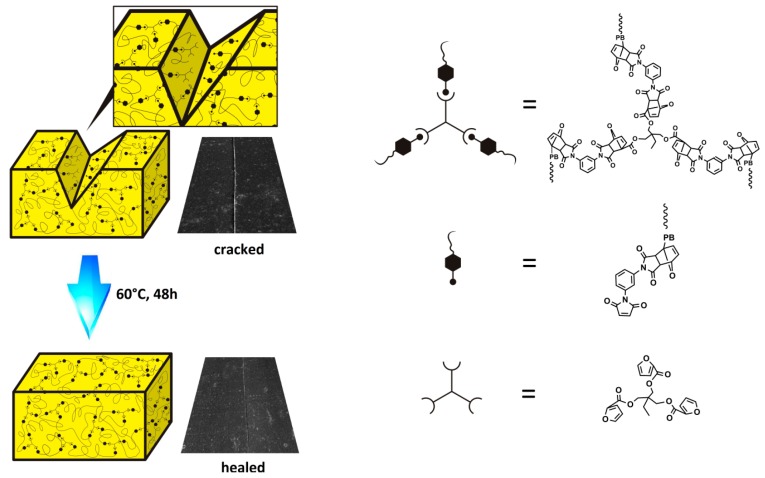
Thermoreversible self-repairing process and scanning electron microscope (SEM) images of FTPB-PDMI film before and after repairing.

In Figure 8, the thermoreversible self-repairing process is illustrated; the crack caused by the mechanical effect destroyed Diels–Alder bonds. However, after the breakage, maleimide and furan groups continued to generate Diels–Alder bonds again after heating at 60 °C for 48 h, which restored the original mechanical properties to a certain extent.

### 3.4. UV-Vis on FTPB-PDMI Film Crack Self-Repairing Progress

**Figure 9:**
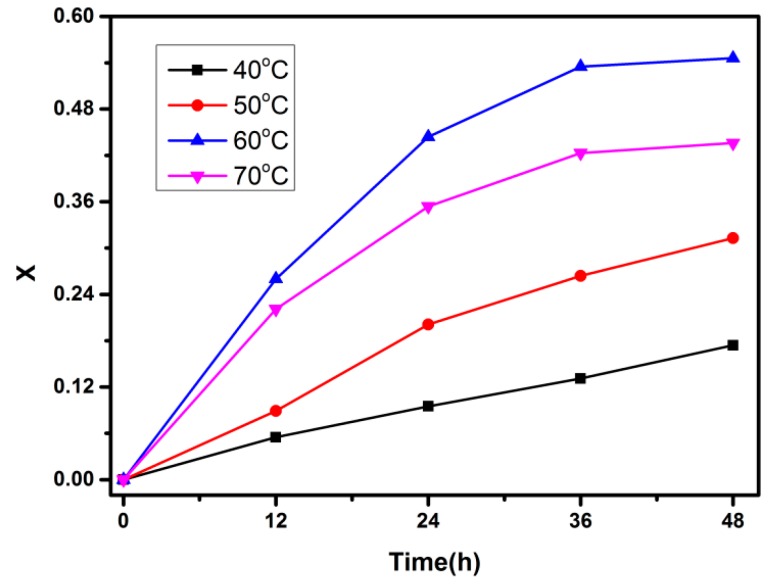
The conversion rate *X* of the FTPB-PDMI varies with time at different reaction temperatures.

In order to study the kinetics of the forward Diels–Alder reaction, UV-Vis test was introduced into this paper. Due to the conjugation effect of ππ* (C=C) and nπ* (C=O) chromophores in the structure of PDMI, PDMI in acetonitrile appears a characteristic absorption peak at 310 nm, and the formation of the Diels–Alder bond between PDMI and FTPB makes this conjugate structure destroyed. Therefore, the UV absorption peak at 310 nm gradually decreased as the reaction progressed, which could measure the reaction kinetics of FTPB-PDMI by the change in the peak. UV-Vis spectrophotometer was used to track the reaction of FTPB-PDMI at different temperatures in acetonitrile and to measure the degree of reaction with the reaction time, and the change of the reaction conversion rate with the reaction time was then calculated (Figure 9).

The reaction kinetics equation generally follows the multi-stage reaction model [20]:

d*x*/d*t* = *k*(1 − *x*)*^n^*. (1)

The conversion rate *x* = 1 − *A_t_*/*A*_0_, *A*_0_, and *A_t_* represents the UV absorption peak of the conjugated double bond in the furan ring before reaction and at the time *t* of reaction at 310 nm, and *n* represents the kinetic order. When the first-order reaction model, *n* = 1, Equation (1) can be written as

ln[1/(1 − *x*)] = *kt*. (2)

When second-order reaction model, *n* = 2, Equation (1) can be written as

1/(1 − *x*) = *kt*. (3)

*K* is the kinetic constant, and the value is related to the reaction system and temperature. Hence, the reaction temperature effect on the kinetic model was studied by using the Arrhenius equation, which is listed as [21]:

*K* = A_1_exp(−*E*_a_/*RT*). (4)

A refers to the pre-exponential factor, *E*_a_ is the activation energy, *R* is the ideal gas constant, and *T* is the absolute temperature. The relationship between ln*k* and 1/*T* can be derived from Equation (4):

ln*k* = A_2_ + (−*E*_a_/*RT*). (5)

**Figure 10:**
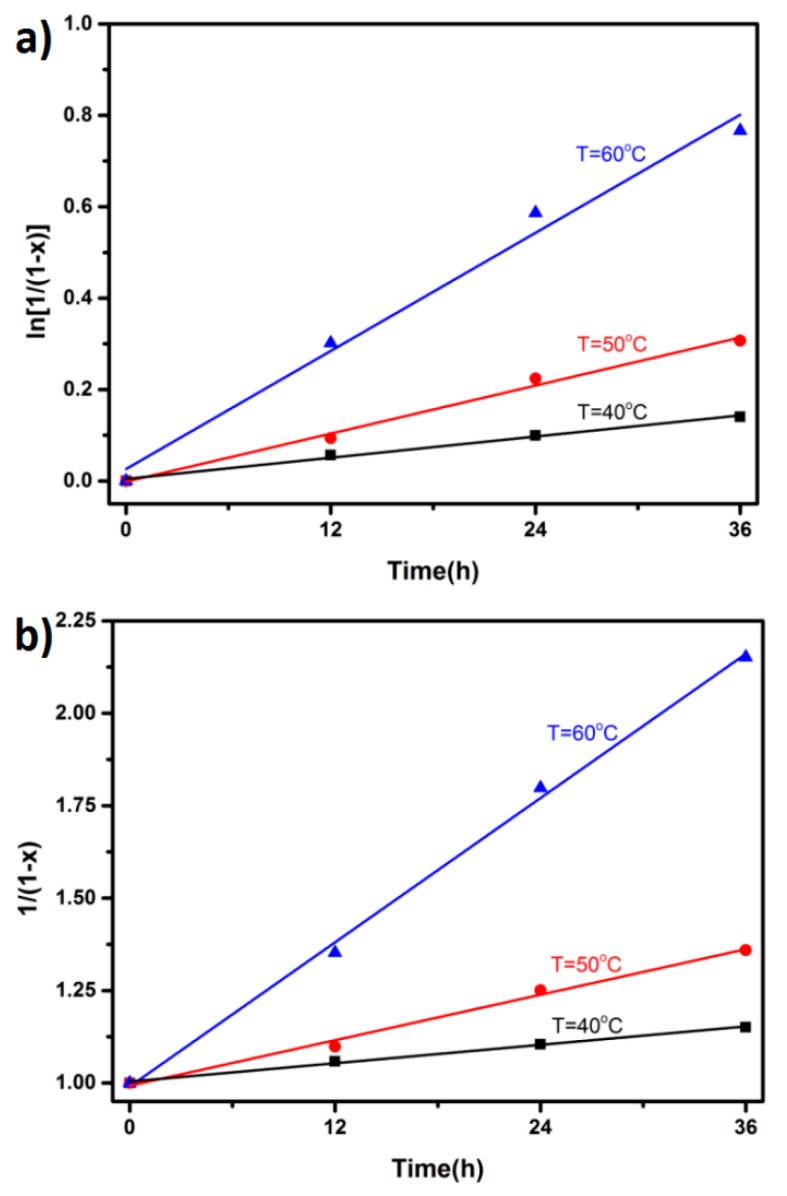
Linear fit of the data is conducted according to (**a**) first-order kinetics; (**b**) second-order kinetics.

The reaction rate increased with time, and the conversion rate at 70 °C was smaller than that at 60 °C (Figure 10). At the same time, it could be deduced that the retro Diels–Alder reaction of FTPB-PDMI was enhanced at 70 °C, which affected the process of reaction and reduced the final conversion. In addition, the reaction rate did not slow down in the first 36 h, so this data was graphed to reduce the error (Figure 10).

Considering that the reverse DA reaction had a significant effect on the kinetics at 70 °C, the value at this temperature was excluded when calculating the forward DA reaction kinetics of the system. According to the calculation equations above, the reaction time t is the abscissa with −ln(1 − *x*) and 1/(1 − *x*) as the ordinate. Due to the first and second reaction kinetics, the model was used to simulate the reaction of FTPB-PDMI at 40, 50, and 60 °C for 48 h. The relationships between reaction conversion and time were calculated, and the results were linearly fitted and shown in Table 2. The activation energy *E*_k_ of the FTPB-PDMI reaction was 86.29 kJ/mol, which was determined by the slope of the line from graphing ln*k* according to 1/*T* obtained by the second-order kinetics model.

## 4. Conclusions

The Diels–Alder reaction could be introduced into HTPB to obtain FTPB, and FTPB/PDMI/TFP system was a self-repairing binder system. When the addition amount of the curing agent was 10%, the tensile strength was 1.76 MPa and the tensile strain was 284%. It was proved that the self-repairing efficiency was based on Diels–Alder reaction. Therefore, the Diels–Alder reaction could provide self-repairing properties for energetic materials such as solid propellants and PBX explosives.

## Figures and Tables

**Table 1:** The tensile test of FPT1, FPT2, and FPT3 before and after self-repairing.

Name	Tensile Strain (%)	Post-Repair Tensile Strain (%)	Tensile Strain Repair Efficiency (%)	Tensile Strength (MPa)	Post-Repair Tensile Strength (MPa)	Tensile Strength Repair Efficiency (%)
FPT1	221	203	91.8	1.59	1.32	83.0
FPT2	284	251	88.4	1.76	1.55	88.0
FPT3	539	471	87.3	0.78	1.00	78.0

**Table 2:** Kinetic rate constants (*k*_1_, *k*_2_) and linear fitting values (*R*) describing the kinetics of model DA reaction.

Kinetic Order	Temperature (°C)	40	50	60
*n* = 1	*k*_1_/min^−1^	0.00396	0.00824	0.02247
	Adj. R-square	0.99891	0.99489	0.99337
*n* = 2	*k*_2_/(min^−1^·mol^−1^·L)	0.00437	0.00973	0.03217
	Adj. R-square	0.99999	0.99986	0.99977
